# The IG- file use to Gauge the Apical Diameter in Endodontics: An *In Vitro* Study

**DOI:** 10.2174/1745017901814010638

**Published:** 2018-09-28

**Authors:** Massimo Amato, Alfredo Iandolo, Giuseppe Pantaleo, Dina Abtellatif, Michele Simeone, Angelo Lizio, Roberto Lo Giudice, Giuseppe Lo Giudice

**Affiliations:** 1 Department of Medicine and Surgery, Salerno University, Salerno, Italy; 2 Department of Neurosciences, Reproductive and Odontostomatological Sciences, Naples Federico II University, Naples, Italy; 3 Department of Endodontics, Faculty of Dentistry, University of Alexandria, Alexandria, Egypt; 4 Department. of Biomedical and Dental Sciences and Morphofunctional Imaging, Messina University, Messina, Italy; 5 Department of Clinical and Experimental Medicine, Messina University, Messina, Italy

**Keywords:** Apical gauging, IG-file, Endodontic treatment, K-NiTi, F1 Universal Protaper, Thermo-plastic gutta-percha

## Abstract

**Aim:**

The aim of this study was to evaluate the efficacy of the IG-file, a new instrument designed for apical diameter gauging.

**Materials and Methods:**

After shaping with F1 Universal Protaper, 60 roots were randomly divided into two groups and assigned to two operators, One Expert in Endodontics (EO) and One Unexpert (UO).

In each sample, after canal curvatures have been detected, the apical diameters were measured with the IG-file and the K-NiTi. The results were compared with the reference value obtained by retrograde apical gauging. The data were statistically analyzed.

**Results:**

Among 60 samples, 10% of errors were recorded when the IG-files were used; in the K-NiTi group the incorrect measurements were 70%.

In both groups (expert and unexpert) the IG-file measurements were more accurate than the K-NiTi (90 *vs* 33 and 90 *vs* 26,7). The differences were statistically significant.

In curved canals, the difference between measurement rates performed with both instruments was statistically significant (85,7% IG-file *vs* 28,6% K-NiTi) as well as for the samples without curvatures (92,3% IG file *vs* 30,8% NiTi file).

In root canals without curvatures overestimation errors in K-NiTi file group are more frequent than underestimation errors. This difference was statistically significant.

**Conclusion:**

A proper gauging of the apical diameter has a key role in endodontic therapy; an incorrect measurement can lead to clinical failures. This “*in vitro*” study highlights that IG-file improves measurement accuracy independently from clinician experience. Furthermore, in curved canals, the IG-file is more accurate than K-NiTi.

## 1 INTRODUCTION

The objective of modern endodontics is to achieve a complete cleaning, shaping and three-dimensional filling of root canal system [1].

The Ni-Ti rotary and manual instruments allowed obtaining more effective and reproducible results in endodontic therapy [2, 3].

Superelasticity of Ni-Ti instruments determines a flexibility and adaptability to canal anatomy useful to maintain a central position, during the canal preparation, and minimize perforation and stripping risks [4]. Moreover, during the shaping step, the fracture risk due to torsional and bending stresses is higher in comparison with stainless-steel instruments [5, 6].

The irrigating solutions with or without activation can improve cleaning of endodontic space [7-13].

At the end of chemical-mechanical shaping of root canals system, the goal is to seal in a three-dimensional way the endodontic space with thermo-plastic gutta-percha.

A 3D obturation of the endodontic system is mandatory to maintain the results obtained with irrigating solution in term of bacterial activity reduction. Indeed, the bacterial persistence in anastomosis, lateral and accessory canals and the complex apical anatomy could determine failure [14].

To obtain a predictable apical sealing it becomes mandatory to detect the apical diameter of the shaped canal, in order to choose the more suitable obturation technique (gutta-percha tips, heat carriers, Thermafil obturators), and to optimize the condensation forces.

Apical gauging is an essential step to obtain certain and reproducible results. Moreover, the knowledge of the right apical diameter would avoid the overfilling of root canals.

The failure of endodontic treatments with overextension of gutta-percha in periodontal space is usually caused by lack of apical sealing associated with inadequate three-dimensional obturation [15].

The overfilling could be the cause of complications like the sliding of obturation materials in maxillary sinus or mandibular canal [16, 17].

The knowledge of the apical diameter allows the clinician to choose a gutta-percha tip that could precisely fill the apical foramen. To obtain a three-dimensional obturation of the endodontic space, is also necessary to choose the correct taper of the gutta-percha cone, so clinician can exploit at the best the condensation forces and the obturation technique, that could hermetically seal the canal system [18, 19].

The instruments normally used for apical gauging are Ni-Ti K-files [18]. In the literature, two main techniques used for apical gauging are reported: Pecking technique and clockwise. At the end of canal shaping phase, the pecking technique consists in drive the K-file at working-length and evaluate its tug-back with a light pressure of forefinger on the file handle.

The clockwise technique provides to drive the K-file until the working-length and to rotate it clockwise for a quarter of turn, and leave it again. If the K-file will return in its original position the measured apical diameter is correct.

K-files are instruments able to shape the root canal, but they show unfavorable features for apical gauging like active tip, transition angle with the first acute coil and presence of coils. Those characteristics could determine an exceeding or lacking measurement of apical diameter.

Indeed, the active tip and the acute transition angle of the first cutting surfaces of K-file could easily let the instrument slip out of the apex.

The IG-file is a new instrument, developed by Dr. Iandolo A., that could be used both for sonic activation of cleaning solutions in the endodontic space and for apical gauging.

The main characteristics of IG-file are: Non-active tip, smooth surface, no cutting angle, inverse taper.

The aim of this research is to test the efficiency of this new instrument to measure the apical diameter.

## 2 MATERIALS AND METHODS

The *in vitro* research was conducted as follows:

Sixty roots of teeth extracted for periodontal reason with fully formed apices and patent foramens were selected. Teeth with root resorption and complex anatomy were excluded from the study [20].

In all samples, the root curved portion was no longer than the apical one-third of the root. Standard access cavities were prepared and patency was confirmed and recorded by inserting a 08 K-file into the root canal until the tip of the file was just visible at the apical foramen.

In order to remove organic debris, the roots were washed with a 0.5% NaOCl solution for 15’ each and then stored in saline solution at 37° C.

Dried sample was processed as indicated:

1. Working length was evaluated with an optical microscope, directly observing the tip of the file at the apical foramen under 10X magnification (OPMI PROergo, Zeiss, Milano, IT) [21].
2. Canal scouting was carried out with K-files 08-10.
3. Glyde path was carried out with Pathfile 1-2-3 (Dentsply, Maillefer, Ballaigues, SWI).
4. Shaping was carried out with Protaper S1-S2 and F1 at WL (Dentsply, Maillefer, Ballaigues, SWI).

After canal shaping, the Apical Diameter (AD) was determined by a retrograde approach with direct observation of IG-file (Iandolo Gauging File) fit with apex.

**Fig. (1):**
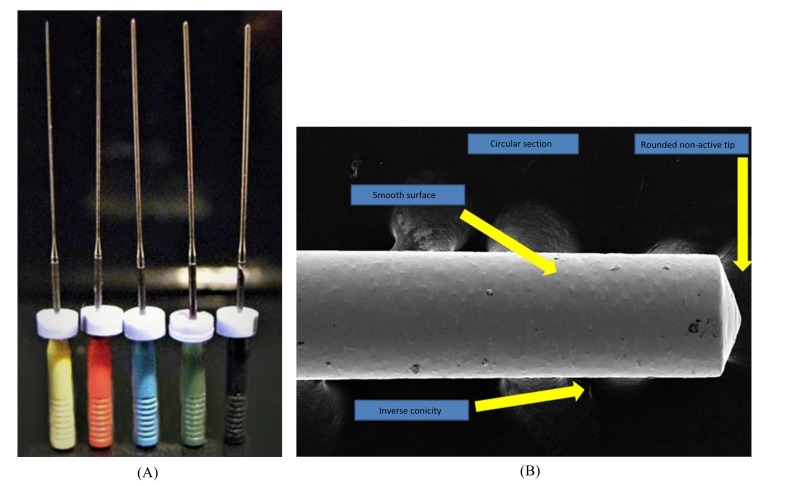
IG-file **a**) ISO diameters from 20 to 40 **b**) Instrument characteristics (SEM analysis).

The IG-file are characterized by a spherical point, a circular section, smooth walls and a 0,003% inverse taper. The instruments are available in ISO diameters from 20 to 60 with 25 and 29 mm lengths [22] (Fig. **1**).

These measurements were performed by an operator that was not involved in the blind part of the study, under 10X magnification.

Two operators, One Expert in endodontics (EO) and One Unexpert (UO) were selected.

The expert operator was selected as dentists graduated from more than 10 years that attended II grade master in endodontic. The unexpert operator was randomly selected from the undergraduate students attending the endodontic department of the Messina University [23].

The samples were randomly divided into two groups and assigned to the two operators. Both operators performed orthograde measurement of apical diameters of 30 samples, randomly selected, with IG-file instruments and, seven days after, measured the same roots with Ni-Ti K-files. To detect measurement errors, the values obtained were compared with the AD values recorded.

The evaluation was followed by a statistical analysis of the results obtained on the experimental groups.

The statistical analysis consisted in the elaboration of categorical variables (operator experience, tooth type and presence of curvatures) frequency and percentage.

Numerical variables were described as average, median and standard deviation.

Statistical analysis was based on experimental questions about measurement accuracy, described in Table **1**.

Results were analyzed with Chi-squared test of Pearson and Fisher’ exact test to individuate variables that influence measurements accuracy (type of instrument, experience of operator and canal curvatures).

## 3 RESULTS

The data analyzed are summarized in Table **2**.

In the IG-file group, 6 errors among 60 samples (10%) were recorded, in the NiTi file group 42 errors among 60 samples (70%) (Table **2**).

The data analysis showed that measurement accuracy for both instruments used was not influenced by the operator experience (*p*=0,999 for IG file e *p*=0,573 for NiTi file) (Table **3**).

In EO group IG-file demonstrated an accuracy of 90% in comparison with 33% of K-NiTi file. This difference was highly statistically significant (*p*-value <0,001) (Table **4**). There was no significant difference between diameter overestimation or underestimation errors for IG-file according to reference values (*p*=0,991). With regard to NiTi file instruments this difference was statistically significant (*p*<0,001) (Table **4**).

In UO group IG File had 90% of reliability in comparison with 26, 7% of NiTi instruments. This difference is statistically significant (*p*-value <0,001) (Table **4**).

In the IG-file group was impossible to compare overestimation and underestimation errors, whereas NiTi file showed 50% of underestimation errors and 23, 3% of overestimation errors. Anyway that comparison was not statistically significant.

In curved canals (21 samples - 35%) IG-file measured apical diameter correctly in 18 cases, while NiTi file only in 6 cases. The rate comparison of exact measurements (85, 7% IG-file *vs* 28,6% K-NiTi) determined a statistically significant *p*-value (*p*<0,001) (Table **5**).

In curved canals there are no significant differences between overestimation and underestimation errors according to a reference value, neither for IG-file (*p*=0,994) nor for NiTi file (*p*=0,053), although the NiTi file group *p*-value is closer to the limit of statistical significance (Table **2**).

In straight canals (39 cases that means 65% of samples) the rate comparison of the right measurement showed a *p*-value statistically significant (*p*< 0,001) (Table **5**).

In straight canals in NiTi instruments group, highly significant differences between overestimation (53,8%) and underestimation (15,4%) errors relating to a reference value *p*=0,000 were recorded, (Table **5**) while the use of IG-file gives only 3 lacking measures. Therefore, it was impossible, in the IG-file group, to carry out the statistical comparison between overestimation and underestimation. As shown by the non-significance of *p*-value relating to both instruments, the operator experience has no influence on correct measurements for both curved (*p*=0,476 IG-file and *p*=0,890 NiTi file) and straight canals (*p*=0,517 IG file and *p*=0,557 NiTi file) (Table **2**).

## 4 DISCUSSION

The knowledge of the canal anatomy and exact apical diameter allows the complete cleaning (complete removal of both organic and non-organic substrates) of endodontic system and an efficient endocanalar obturation.

A correct apical gauging allows the operator to choose the most adequate obturation materials (gutta-percha tips, heat carriers, Thermafil obturators), to fill the canal, to optimize condensation forces and avoid, in the apical area, inadequate seal e/o overfilling [17].

The Ni-Ti K-files are commonly used to gauge the apex. K-files was designed to shape the canal, and so presents some unfavorable features if used to gauge the apex: Active tip, acute transitional angle of the cutting surface. Those characteristics, in our opinion, could determine an exceeding or lacking measurement of the apex gauging.

The blade design and the taper could determine a lacking measure, while the active tip and the acute transitional angle could determine an exceeding one. These features could be the cause of slipping of the instruments over the apex [18].

The IG-file could be used for apical gauging. The main characteristics are a non-active tip, smooth surface, and absence of cutting angles and inverse taper. The inverse taper, indeed, allows the IG-file to have a glide path exactly until the apex and have a correct apex gauging [22].

While the accuracy of working length determination is aided by electronic apex locators and radiographs, the apical gauging depends on operator technical skills. The operator experience is also necessary to detect instrument binding into the canal walls and curved canal anatomy.

Considering the bias that is always present in a clinical evaluation, we decided to structure the study following a protocol used as gold standard to evaluate the precision of an endodontic instrument, represented as *in vitro* evaluation carried out with an optical microscopy assessment of the working length. Moreover, clinical study that assesses the precision of the instrument has already been reported in the literature [22].

Comparison of efficiency of apical gauging done with two different instruments as analysed in our study, proves how instrument’s type takes a great relevance in apical size determination for both, experienced and inexperienced operator.

Furthermore, in experienced group no statistical difference was found between exceed or deficiency measurement, relative to IG-file unlike NiTi instruments.

Instead, in an inexperienced group, it was impossible to point out any difference between such errors for IG-file and there was no significant discrepancy in regard to NiTi file. In curved root canals the type of instrument assumes a great importance in proper canal gauging and IG-file seems to be a more accurate.

Evenly, in the straight canal, the type of instrument has the same importance in correct apical diameter determination compared to a reference value. Particularly, K-NiTi tends to a deficiency measurement.

Clinician experience seems to have no influence in apical gauging for both instruments, K-NiTi and IG-file.

## CONCLUSION

In order to improve the long-term results of root canal treatment, the goal is a complete shaping of the canal system with chemical and mechanical approach.

The next step is obturation and apical sealing, to inactivate biologically the bacterial invasion of endodontic space. Therefore, the apical gauging is necessary for a successful treatment. It is evident how an expert operator could correctly measure the apical diameter with the standard instruments and techniques. The statistical analysis of our data shows that the type of instrument takes a great relevance for the accuracy of executed measurements related to a reference value in both groups, expert and unexpert operators.

This study suggests that the use of IG-file could improve the precision of the apical gauging measurement and the long-term results of the endodontic treatment.

## Figures and Tables

**Table 1:** Experimental question.

Aggregate samples	Instruments design effect (IG file K-NiTI) on apical gauging
	Operator experience effect on apical gauging correct measurement
	Exceeding or lacking apex gauging (IG file EO *Vs* UO)
	Exceeding or lacking apex gauging (K-NiTI EO *Vs* UO)
With or without canal curvature	Measurements accuracy (IG file *Vs* K-NiTi file)
	Exceeding or lacking apex gauging (IG file -NiTi file)
	Operator experience effect on apical gauging correct measurement
	Instruments design effect (IG file K-NiTI) on apical gauging

**Table 2:** Experimental groups.

-	**Tooth**	**Apical** **Diameter**	**Canal Curvature**	**IG-NiTi Measurement**	**NiTi File Measurement**
**Expert operator**					
1	Low. Prem.	30	–	30	25
2	Low. Mol.	30	Yes	30	25
3	Low. Mol.	20	Yes	20	20
4	Low. Mol.	25	Yes	25	20
5	Upp. Mol.	40	-	40	30
6	Upp. Mol.	35	-	30	30
7	Upp. Mol.	25	-	25	25
8	Low. Mol.	25	-	25	20
9	Low. Mol.	30	-	30	25
10	Low. Can	30	Yes	30	20
11	Low. Prem.	30	-	30	25
12	Upp. Inc.	30	-	30	25
13	Upp. Inc.	25	-	25	25
14	Upp. Inc.	25	Yes	25	20
15	Low. Prem.	35	-	35	35
16	Low. Inc.	25	-	25	20
17	Low. Prem.	35	-	35	30
18	Low. Inc.	30	Yes	30	25
19	Low. Inc.	35	-	35	30
20	Low. Prem.	30	-	30	30
21	Upp. Mol.	25	Yes	25	30
22	Upp. Mol.	25	-	25	25
23	Upp. Mol.	30	Yes	35	30
24	Upp. Mol.	30	-	30	25
25	Upp. Mol.	20	Yes	20	20
26	Upp. Mol.	25	Yes	30	30
27	Low. Can.	20	-	20	25
28	Upp. Mol.	25	-	25	25
29	Upp. Mol.	25	-	25	25
30	Upp. Mol.	25	-	25	20
**Unexpert Operator**					
1	Upp. Prem.	30	-	30	25
2	Upp. Inc.	30	Yes	30	25
3	Low. Inc	30	Yes	30	20
4	Upp. Prem.	25	-	25	30
5	Upp. Mol.	35	-	35	25
6	Upp. Mol.	35	-	30	25
7	Upp. Mol.	40	Yes	40	25
8	Upp. Mol.	30	-	30	20
9	Upp. Mol.	35	Yes	30	20
10	Upp. Mol.	40	-	40	25
11	Upp. Mol.	35	-	35	25
12	Upp. Mol.	30	-	30	20
13	Upp. Inc.	20	Yes	20	20
14	Upp. Inc.	40	-	40	25
15	Upp. Mol.	25	Yes	25	20
16	Low. Inc.	25	Yes	25	25
17	Low. Prem.	30	-	30	30
18	Low. Inc.	30	Yes	30	25
19	Upp. Mol.	20	-	20	20
20	Upp. Mol.	25	-	20	30
21	Upp. Mol.	30	-	30	25
22	Upp. Mol.	25	-	25	30
23	Upp. Mol.	25	-	25	30
24	Upp. Mol.	25	-	25	30
25	Low. Mol.	30	Yes	30	35
26	Upp. Can.	25	-	25	25
27	Upp. Mol.	20	Yes	20	25
28	Low. Mol.	25	Yes	25	25
29	Low. Prem.	25	-	25	25
30	Low. Prem.	25	-	25	25

**Table 3:** Statistical analysis of the experience impact.

-	**IG-File**			**K-NiTi**		
-	Asymp. Sig. (2-sided)	Exact Sig. (2-sided)	Exact Sig. (1-sided)	Asymp. Sig. (2-sided)	Exact Sig. (2-sided)	Exact Sig. (1-sided)
Pearson's chi-squared test	.999	-	-	.573	-	-
Continuity correction	1.000	-	-	.778	-	-
Likelihood-ratio	1.000	-	-	.573	-	-
Fisher's exact test	-	1.000	.665	-	.779	.389
Linear by-linear association	1.000	-	-	.576	-	-

**Table 4:** Analysis of the apical measurement.

-	Expert		Unexpert	
-	IG-File	K-NiTi	IG-File	K-NiTi
Correct measurement	27 (90%)	10 (33.3%)	27 (90%)	8 (26.7%)
Overestimation	2 (6.7%)	3 (10%)	--	7(23.3%)
Underestimation	1 (3.3%)	17 (56.7%)	3 (10%)	15(50%)

**Table 5:** Analysis of canal curvature effect on apical measurement.

-	Curved Canals		Straight Canals	
	IG-File	K-NiTi	IG-File	K-NiTi
Count	18	6	36	12
Percent	85.7	28.6	92,3	30,8
Stand. Error	0.152		0.110	
P value	0.000		0.000	
